# Cycloartane Triterpenoids from *Euphorbia Macrostegia* with their Cytotoxicity against MDA-MB48 and MCF-7 Cancer Cell Lines 

**Published:** 2014

**Authors:** Somayeh Baniadam, Mohammad Reza Rahiminejad, Mustafa Ghannadian, Hojjatollah Saeidi, Abdul Majid Ayatollahi, Mahmoud Aghaei

**Affiliations:** a *Isfahan Pharmaceutical Sciences Research Center, Isfahan University of Medical Sciences, Isfahan, I.R. Iran. *; b *Department of Biology, Faculty of Science, University of Isfahan, Isfahan, I.R. Iran.*; c *Department of Pharmacognosy, School of Pharmacy, Isfahan University of Medical Sciences, Isfahan, I.R. Iran.*; d *Phytochemistry Research Center,Department of Pharmacognosy, School of Pharmacy, Shahid Beheshti University of Medical Sciences, Tehran, Iran.*; e *Department of Clinical Biochemistry, School of Pharmacy, Isfahan University of Medical Sciences, Isfahan, I.R. Iran.*

**Keywords:** *Euphorboa macrostegia*, Cycloartane, Cytotoxicity, MDA-MB468, MCF-7

## Abstract

The dried plant was extracted with dichloromethane and after defatting with hexane, transferred repeatedly on silica columns using dichloromethane-hexane and ethyl acetate-hexane as mobile phases. Finally the fractions were purified by high performance liquid chromatography using a Pack-Sil column and hexane: Ethyl acetate as mobile phase. The structures of the isolated compounds included: cycloart-25-ene-3β, 24-diol (1), cycloart-23(Z)-ene-3β, 25-diol (2), cycloart-23(E)-ene-3β, 25-diol (3), and 24-methylene-cycloart-3β-ol (4) were elucidated by ^13^C- and ^1^H-NMR as well as IR and by the aid of mass fragmentation pattern and comparing with the literature. The biological effects of the compounds were done by the MTT assay on two different cancer cell lines including MDA-MB48 and MCF-7. Among these compounds, cycloart-23(E)-ene-3β,25-diol (3) was the most active compound on MDA-MB468 cell line (LD_50 _= 2.05 μgmL^− 1^ ) and cycloart-23(Z)-ene-3β, 25-diol (2) was the most active compound on MCF-7 cell line (LD_50 _= 5.4 μgmL^− 1^).

## Introduction

The incidence of cancer in human populations and the increasing need for anti-cancer drugs on the one hand and discovery of effective anti-cancer drugs, such as taxol, vincristine and vinblastin from plants. *E.macrostegia *as one of the endemic plants to Iran is the subject of this investigation. *Euphorbia macrostegia *(Persian wood spurge), belongs to the family Euphorbiaceae distributed mostly in central and west parts of Iran. Persian wood spurge is similar to the wood spurge (*Euphorbia amygdaloides*) and a rare species native of semi-moist woods from south-eastern Europe through Asia Minor. In the Iranian traditional medicine, latex is used to treat warts. Despite their toxicity, the uses of *Euphorbia* species in traditional medicine in many parts of the world have a long history. They are used to treat inflammations and tumours (1). Previous investigation on the cytotoxicity assessment of *E. macrostegia *(2), has showed LD_50_ values of 200, 425, and 390 μgmL^− 1^ for dichloromethane, ethyl acetate and acetone fractions, respectively while other fractions, remarked as noncytotoxic. Therefore, based on previous studies on cytotoxiciy effects of *E. macrostegia *and its fractions, the authors decided to investigate phytochemical contents of the dichloromethane extract of this plant as the most active fraction.

**Figure 1:**
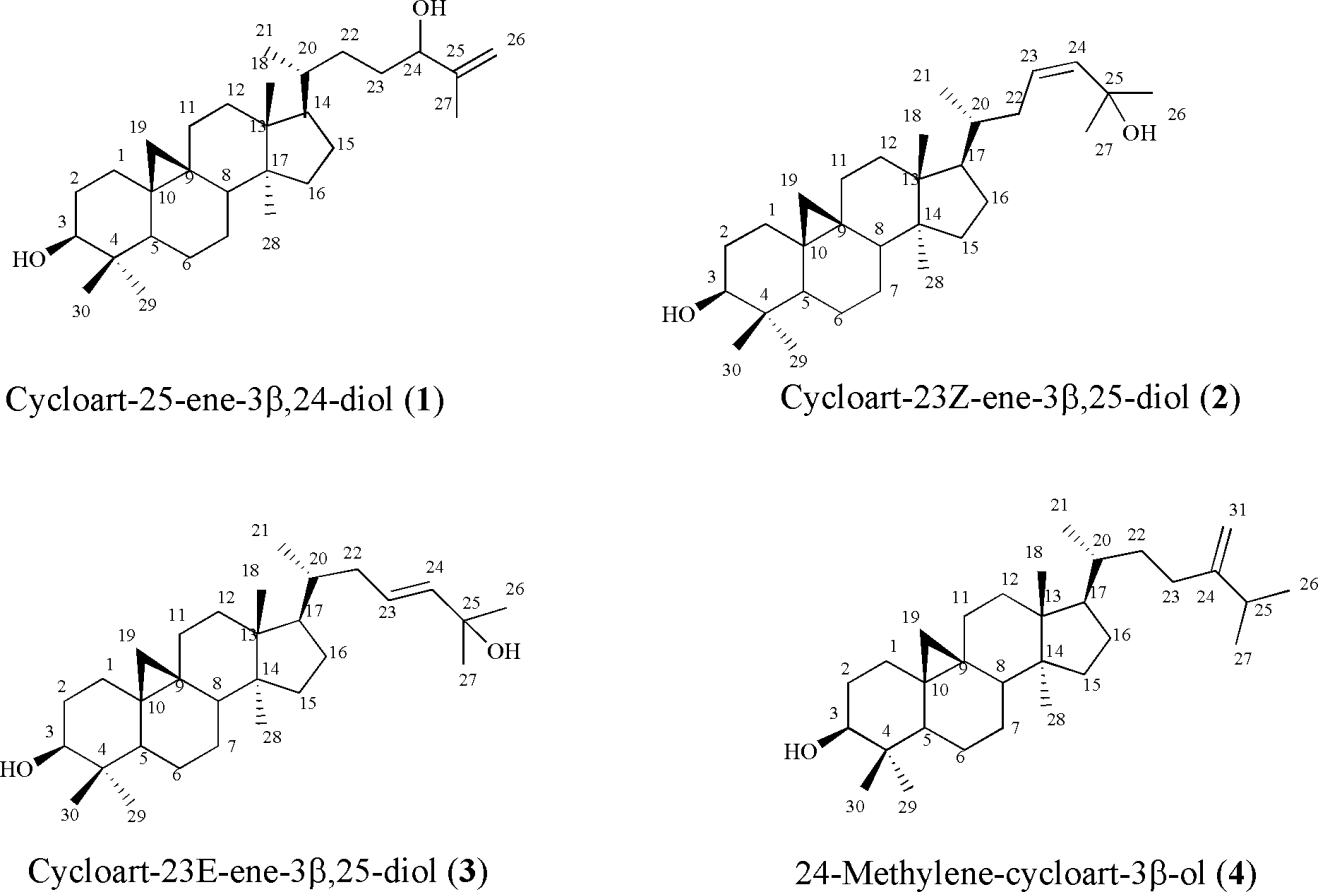
Triterpenoids from *Euphorbia macrostegia*

## Result and Discussion

Compound 1, white crystals, showed the molecular formula of C_30_H_50_O_2_ based on EI-MS *m/z* 442 and number and multiplicity of ^13^C-NMR spectra. The six-degree of unsaturation and the ^13^C-NMR data (Table 1), suggested the presence of one double bond and, therefore, a pentacyclic skeleton. EI-MS fragmentation pattern, supported *m/z* 355 and 302, typical ions of 4,4' dimethyl 9:19 cycloesterols (4). ^1^H-NMR revealed a pair of doublets in the up-field area 0.57, 0.36 (each ^1^H, d, *J *= 4.0 Hz, H-19a, b), characteristic of cycloartane cyclopropane ring (4), one secondary methyl group at 0.90 (3H, d, *J *= 6.4 Hz, H-21), and five singlet methyls at δ_H_ 0.83 (3H, s, H-29), 0.91 (3H, s, H-28), 0.98 (3H, s, H-18), 0.99 (3H, s, H-30), and at 1.73 (3H, s, Me-27). Two double doublet protons at δ_H_ 3.30 (^1^H, dd, *J *= 4.4, 10.8 Hz, H-3), and δ_H _4.03 (1H, t, *J *= 5.8 Hz, H-24) revealed presence of two carbinolic protons and a pair of olefinic protons at δ_H_ 4.95 and 4.86 (each ^1^H, brs, H-26) suggested a terminal methylene. Downfield chemical shift of one singlet methyl proton at δ_H_ 1.73 (H-27) of the side chain atoms was in accordance with the quaternary olefinic group on C-25 at δ_C_ 128.8. As Ayatollahi and coworkers described EI-MS fragmentation pattern of cycloartanes (4), presence of monounsaturated side chain was also confirmed by the *m/z* 315 and 297 in EI-MS. In addition, *m/z* 381 together with 355 [M-H_2_O-C_5_H_9_]^+^ fragments due to the elimination of parts of side chain during a Mc Lafferty process, inferred presence of one hydroxyl in side-chain. Regarding to these findings, and literature data (4), compound 1 identified as cycloart-25-en-3 β, 24-diol. It is also found in other *Euphorbia* species like *E. aellenii*** (4), *E****. heteradena* (5) and *E. sessiliflora*** (**6**).**

Compound 2, and 3 showed the molecular formula of C_30_H_50_O_2_ based on positive EI- MS *m/z* 442 and in accordance with their number and the multiplicity of ^13^C-NMR spectra (BB and DEPT). Their ^1^H-NMR revealed six tertiary singlet methyls, one secondary methyl group, and a pair of doublets in the up-field area characteristic of cycloartane cyclopropane ring and one carbinolic proton related to 3(β)-OH group. In compound 2, in olefinic pair protons, δ_H_ 4.94 (^1^H, brs, H-24) showed low coupling constants with at δ_H_ 4.96 (^1^H, m, H-23) due to their cis orientation while in compound **3**, olefinic pair protons at δ_H_ 5.72 (^1^H, ddd, *J *= 15.6, 8.4, 6.0 Hz, H-23) and 5.54 (^1^H, d, *J *= 15.6 Hz, H-24) with large coupling constant (*J *= 15.6 Hz) allowed assignment of trans geometry to the Δ23(24). In both compounds, downfield chemical shifts of two singlet methyl protons (Me-26, and Me-27) of the side chain atoms were in accordance with the second hydroxyl group on C-25 at δ_C_ 70.8 and 68.2, respectively. Therefore, based on aforementioned data and complete agreements of ^13^C- and ^1^H-NMR with other reported data in literature (7; 8), compound 2 and 3 were identified as cycloart-23Z-ene-3β, 25-diol and cycloart-23E-ene-3β, 25-diol (Figure 1). They are also reported in *Euphorbia spinidens* (9), *E. rigida* (10), and *E. humifusa* (11).

Compound 4, showed the molecular formula of C_31_H_52_O based on EI-MS *m/z* 440 and number and multiplicity of ^13^C-NMR spectra. The six-degree of unsaturation and the ^13^C-NMR data (Table 1), suggested the presence of one double bond and consequently five rings in the molecule. The ^13^C-NMR data (BB and DEPT), encompassed thirty-one carbons.^1^H-NMR revealed a pair of doublets in the up-field area at δ_H_ 0.30 and 0.53 (*J *= 4.25 Hz) characteristic of cycloartanes, four singlet methyls at δ_H_ 0.83 (3H, s, H-29), 0.91 (3H, s, H-28), and 0.99 (2× 3H, s, H-18, H-30) together with three secondary methyls. A doublet of doublet proton at δ_H_ 3.31, indicative of a carbinolic group, and one pair of olefinic protons δ_H_ 4.74, and 4.69 (each ^1^H, bs, H-31a, b) related to exocyclic terminal methylene. According to the literarture and these data, compound 4 was determined as 24-methylene-cycloartan-3β-ol (4). It was found in other spurge species like *E. rigida* (10), and *E. aellenii*** (**4**).**

Using MTT assay on two different cancer cell lines (3,12-13), the biological effects of the compounds (1-4) on two different cancer cell lines including MDA-MB48 and MCF-7 showed LD_50_ values of 102.3, 34.0, 2.05, and 53.8 μgmL^−1^ on MDA-MB468 cell line, and LD_50_ values of 88.3, 5.4, 8.9, and 127.3 μgmL^− 1^ on MCF-7 cell line, respectively. Among these compounds, cycloart-23(E)-ene-3β,25-diol (3) was the most active compound on MDA-MB468 cell line (LD_50_ = 2.05 μgmL^− 1^ ) and cycloart-23(Z)-ene-3β,25-diol (2) was the most active compound on MCF-7 cell line (LD_50_ = 5.4 μgmL^− 1^ ).

The potent cytotoxicity observed by compound 2 and 3 with double bound on C-23 suggested that the cytotoxicity activities of these compounds are related to the position of the olefinic or the hydroxyl group on side chain.

**Figure 2:**
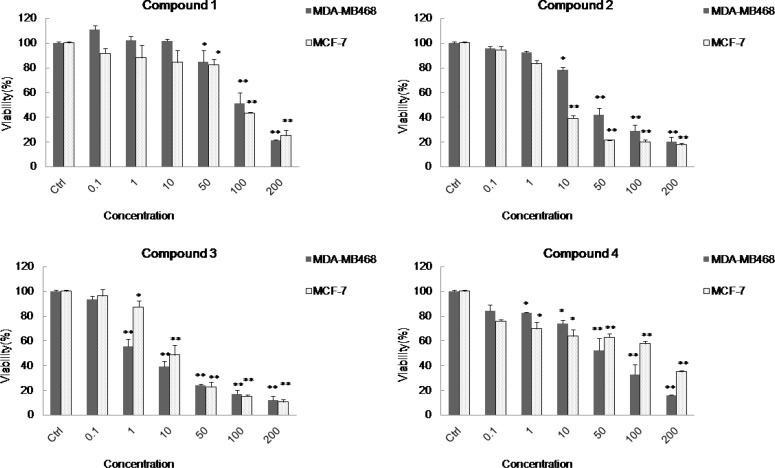
Cytotoxicity effects of the cycloartanes (1-4) in *Euphorbia macrostegia* on two cancer cell lines MDA-MB48 and MCF-7 . In this panel the cytotoxicity tests were presented on two different cancer cell lines including MDA-MB48 and MCF-7 in the presence of different concentrations (0.1, 1, 10, 50, 100 and 200 μg/mL) of cycloart-25-ene-3β,24-diol (1), cycloart-23(Z)-ene-3β,25-diol (2), cycloart-23(E)-ene-3β, 25-diol (3), and 24-methylene-cycloart-3β-ol (4), and control cells which were not treated (set to 100%). For statistical significance one-way ANOVA was used to analyze the differences between each sample and control (*P < 0.05, **P < 0.01).

By the literature, cycloartanes isolated from *Euphorbia* species showed also apoptosis induction on mouse lymphoma cells (14). Cycloart-25-en-3(β), 24-diol and 24-methylene-cycloartan-3(β)-ol (compound 1 and 4) presented antiproliferated activity on human peripheral blood lymphocytes (4). Cycloartanes were also reported for other biological activities like immunomodulatory effects like positive effect on Th1 cytokine release (IL-2 and IFN-γ), and suppression on Th2 cytokine production (IL-4) (15), inhibition of 11β-hydroxysteroid dehydrogenases (11β-HSD1 and 11β-HSD2) as a strategy for reducing glucocorticoid action on insulin resistance in type 2 diabetes mellitus and metabolic syndrome (16,17), or stimulating GLP-1 amide secretion in streptozotocin-nicotinamide induced diabetic Sprague Dawley rats (18). Therefore, interesting properties of cycloartanes, especially their antiproliferative effects, candidate them as investigational lead compounds in cancer research.

## Figures and Tables

**Table 1:** 13C-NMR chemical shifts of the triterpenoids from *Euphorbia macrostegia*.

**C**	**1**	**2**	**3**	**4**	**C**	**1**	**2**	**3**	**4**
**1**	31.9	32.0	32.0	32.0	**16**	26.5	26.4	26.4	26.5
**2**	30.4	30.4	30.4	30.4	**17**	52.2	52.0	52.1	52.3
**3**	78.9	78.8	78.8	78.9	**18**	18.0	19.3	18.1	18.1
**4**	40.5	40.5	40.5	40.5	**19**	29.9	30.1	29.9	29.9
**5**	47.1	47.7	47.1	47.1	**20**	35.9	36.4	36.3	36.4
**6**	21.1	21.1	21.1	21.1	**21**	18.3	18.3	18.4	18.3
**7**	28.1	28.1	28.1	28.2	**22**	32.0	39.1	39.4	35.0
**8**	48.0	48.0	48.0	48.0	**23**	31.5	125.6	130.8	31.3
**9**	20.1	20.0	20.0	20.0	**24**	76.7	139.3	134.4	156.9
**10**	26.1	26.1	26.0	25.8	**25**	128.8	70.8	68.2	33.8
**11**	26.0	26.0	26.0	26.0	**26**	11.4	29.9	24.4	22.0
**12**	32.9	32.8	32.8	32.9	**27**	17.2	29.9	24.3	21.9
**13**	45.3	45.3	45.3	45.3	**28**	19.3	19.3	19.3	19.3
**14**	48.7	48.8	48.8	48.8	**29**	14.0	14.0	14.0	14.0
**15**	35.6	35.6	35.6	35.9	**30**	25.4	25.5	25.4	25.5

## Exprerimental

*General experimental procedures*

The NMR spectra were recorded on a Bruker Avance AV 400, using CDCl_3_ as solvent. HPLC was carried out on a waters 515 using a YMC-Pack-Sil column (250 × 20 mm *i.d*.) and hexane: EtOAc as mobile phase. Chromatographic materials were silica gel (Merck Co., Germany). Thin layer chromatography detection was achieved by spraying the silica gel plates with cerium sulfate in 10% aq.H_2_SO_4_, followed by heating.

*Plant material*

Plant material was collected from Margoon water fall with elevation of 2130 m A.S.L. located in Yasooj, a city of Kohkilouyeh Va Boyer Ahmad province at Iran. It was identified by Department of Biology, Faculty of Science at University of Isfahan and a voucher specimen (#3340) was deposited in the herbarium of the Isfahan University (Iran).

*Extraction and isolation*

- The air-dried plant material (2 Kg) was macerated with chloroform (20 L×3) at room temperature for 5 days. Filtration and *in vacu*o concentration resulted in a green gum (110 g), which was subjected on silica gel CC (hexane/dichloromethane, 0→100) to several fractions: Fr 1-Fr 5. Inferred from TLC and ^1^H-NMR, fraction Fr.1 and Fr.2 contained alkanes and fats, Fr.3 containing beta-sitosterol and fraction Fr. 4 and Fr.5 triterpenes. Fr.4 and Fr.5 were chromatographed on another normal column (hexane/acetone, 0→20). Finally triterpenes was further purified on HPLC using YMC-Pak-Sil column (250 × 20 mm) and hexane:ethylacetate (80:20) as mobile phase to yield compounds 1-4.

*Cycloart-25-ene-3β,24-diol (1)*

White crystals; MW(g/mol): 442; yield: 0.0010%; ^1^H-NMR (CDCI3, 400 MHz): δ_H_ 4.95, 4.86 (each ^1^H, brs, H-26), 4.03 (^1^H, t, *J *= 5.8 Hz, H-24), 3.30 (^1^H, dd, *J *= 4.4, 10.8 Hz, H-3), 1.73 (3H, s, H-27), 0.99 (3H, s, H-30), 0.98 (3H, s, H-18), 0.91 (3H, s, H-28), 0.90 (3H, d, *J *= 6.4 Hz, H-21), 0.83 (3H, s, H-29), 0.57, 0.36 (each ^1^H, d, *J *= 4.0 Hz, H-19a, b); ^13^C-NMR data: see Table 1. EIMS *m/z*: 442 (5), 427 (5), 424 (12), 409 (17), 381 (8), 355 (2), 315 (7), 302 (21), 297 (8), 203 (28), 175 (59), 43 (100).

*Cycloart-23Z-ene-3β,25-diol (2)*

White crystals; MW(g/mol): 442; yield: 0.0004%; ^1^H-NMR (CDCI_3_, 400 MHz): δ_H_ 4.96 (^1^H, m, H-23), 4.94 (^1^H, brs, H-24), 3.22 (^1^H, dd, *J *= 4.4, 11.2 Hz, H-3), 1.27 (3H, s, H-26), 1.26 (3H, s, H-27), 0.90 (2× 3H, s, H-18, H-29), 0.81 (3H, s, H-30), 0.79 (3H, d, *J *= 6.4 Hz, H-21), 0.74 (3H, s, H-28), 0.49, 0.25 (each ^1^H, d, *J *= 4.4 Hz, H-19a, b) ); ^13^C-NMR data: see Table 1; EIMS *m/z*: 442 (3), 427 (6), 425 (15), 409 (5), 383 (3), 363 (5), 357 (3), 326 (16), 315 (6), 302 (13), 300 (30), 297 (9), 269 (7), 175 (52), 43 (100).

*Cycloart-23E-ene-3β,25-diol (3)*

White crystals; MW(g/mol): 442; yield: 0.0015%; ^1^H-NMR (CDCI_3_, 400 MHz): δ_H_ 5.72 (^1^H, ddd, *J *= 15.6, 8.4, 6.0 Hz, H-23), 5.54 (^1^H, d, *J *= 15.6 Hz, H-24), 3.30 (^1^H, dd, *J *= 4.4, 10.8 Hz, H-3), 1.37 (2 × 3H, s, H-26, H-27), 1.0 (3H, s, H-29), 0.99 (3H, s, H-18), 0.91 (3H, s, H-30), 0.89 (3H, d, *J *= 6.4 Hz, H-21), 0.83 (3H, s, H-28), 0.58, 0.36 (each ^1^H, d, *J *= 4.0 Hz, H-19a, b) ); ^13^C-NMR data: see Table 1. EIMS *m/z*: 442 (3), 424 (10), 409 (14), 315 (6), 302 (9), 297 (10), 255 (16), 203 (36), 187 (45), 175 (60), 145 (67), 43 (100).

*Cycloart-24-en-3β-ol (4)*

White crystals; MW(g/mol): 440; yield: 0.0005%; ^1^H-NMR (CDCI_3_, 400 MHz): δ_H_ 4.73, 4.71 (each ^1^H, bs, H-31a,b), 3.31 (^1^H, dd, *J *= 4.4, 11.2 Hz, H-3), 1.05 (3H, d, *J *= 6.4, H-27), 1.04 (3H, d, *J *= 6.8, H-26), 0.99 (2× 3H, s, H-18, H-30), 0.91 (3H, s, H-28), 0.90 (3H, d, *J *= 9.2 Hz, H-21), 0.83 (3H, s, H-29), 0.57, 0.36 (each ^1^H, d, *J *= 4.0 Hz, H-19a, b) ); ^13^C-NMR data: see Table 1; EIMS *m/z*: 440 (7), 425 (12), 407 (21), 315 (8), 300 (19), 297 (11), 286 (28), 203 (55), 175 (72), 69 (100).

*Cell culture*

MCF-7 and MDA-MB468 human breast cancer cell lines were obtained from Pasteur Institute of Iran. The cell lines were grown adherently in RPMI-1640 media supplemented with10% fetal calf serum, 100 U/mL penicillin and 100 µg/mL streptomycin at 37 °C in 5% CO_2_/ 95% air**.**

*MTT viability assay*

Cell viability was determined by 3-(4, 5-dimethylthiazol-2-yl)-2, 5-diphenyltetrazolium bromide (MTT) assay. The MCF-7 and MDA-MB468 cells were seeded at 5 × 10^3^ cells/well in 5% CO_2_ at 37 °C in RPMI medium (containing 10% FBS, 100 units⁄mL penicillin and 100 μg⁄mL streptomycin) in 96-well plates. After incubation overnight to allow for cell attachment, the RPMI medium in each well was replaced with by media containing various concentrations of compounds and incubated for 48 h. Afterwards, 20 μL of MTT (5 mg/mL in PBS) was added to each well and the cells were incubated for another 4 h at 37 °C. The supernatants were then aspirated carefully and 200 μL of dimethyl sulfoxide (DMSO) was added to each well. The plates were shaken for an additional 10 min and the absorbance values were read by the microplate reader (Bio-Rad, Hercules, CA, USA) at 570 nm. Cell viability was calculated as a percentage using the formula: (mean OD of treated cells /mean OD of control cells) ×100. The results expressed as percent of control cells which were not treated (3).

*Statistical analysis*

All samples were presented as mean ± SD for three measurements. Significance was attributed to p-values (P < 0.05) and the probability values obtained by the student t-test between sample and control data.
